# Transferability of N-terminal mutations of pyrrolysyl-tRNA synthetase in one species to that in another species on unnatural amino acid incorporation efficiency

**DOI:** 10.1007/s00726-020-02927-z

**Published:** 2020-12-17

**Authors:** Thomas L. Williams, Debra J. Iskandar, Alexander R. Nödling, Yurong Tan, Louis Y. P. Luk, Yu-Hsuan Tsai

**Affiliations:** School of Chemistry, Cardiff University, Cardiff, CF10 3AT UK

**Keywords:** Genetic code expansion, Unnatural amino acid, Non-canonical amino acid, Pyrrolysyl-tRNA synthetase (PylRS)

## Abstract

Genetic code expansion is a powerful technique for site-specific incorporation of an unnatural amino acid into a protein of interest. This technique relies on an orthogonal aminoacyl-tRNA synthetase/tRNA pair and has enabled incorporation of over 100 different unnatural amino acids into ribosomally synthesized proteins in cells. Pyrrolysyl-tRNA synthetase (PylRS) and its cognate tRNA from *Methanosarcina* species are arguably the most widely used orthogonal pair. Here, we investigated whether beneficial effect in unnatural amino acid incorporation caused by N-terminal mutations in PylRS of one species is transferable to PylRS of another species. It was shown that conserved mutations on the N-terminal domain of MmPylRS improved the unnatural amino acid incorporation efficiency up to five folds. As MbPylRS shares high sequence identity to MmPylRS, and the two homologs are often used interchangeably, we examined incorporation of five unnatural amino acids by four MbPylRS variants at two temperatures. Our results indicate that the beneficial N-terminal mutations in MmPylRS did not improve unnatural amino acid incorporation efficiency by MbPylRS. Knowledge from this work contributes to our understanding of PylRS homologs which are needed to improve the technique of genetic code expansion in the future.

**Supplementary Information:**

The online version contains supplementary material available at 10.1007/s00726-020-02927-z.

## Introduction

The ability to genetically introduce an unnatural (i.e., non-canonical) amino acid with unique chemical and physical properties into a defined position of a target protein has provided a new avenue to investigate protein function (Brown et al. 2018; Chin 2017; Nodling et al. 2019; Young and Schultz 2018). This approach known as genetic code expansion relies on an orthogonal aminoacyl-tRNA synthetase/tRNA pair to direct the site-specific incorporation of an unnatural amino acid in response to a blank codon. The amber stop codon (UAG) is usually chosen as the blank codon because it does not encode a canonical amino acid and is the least used codon in most organisms (Athey et al. 2017). Here, it is critical that the orthogonal synthetase only acylates the orthogonal tRNA with the designated unnatural amino acid, and neither the orthogonal tRNA nor the unnatural amino acid is a substrate of endogenous synthetases. The pyrrolysyl-tRNA synthetase (PylRS) from archaea *Methanosarcina barkeri* (MbPylRS) or *Methanosarcina mazei* (MmPylRS) and its cognate tRNA (Pyl tRNA) are arguably the most widely used orthogonal pair for genetic code expansion in bacteria and eukaryotes (Brown et al. 2018; Chin 2017; Nodling et al. 2019). The two organisms have identical sequences for Pyl tRNA (Srinivasan et al. 2002; Suzuki et al. 2017) that naturally decodes the amber codon. MbPylRS and MmPylRS also have high sequence identity (74%). While wild-type MbPylRS and MmPylRS can recognize some unnatural amino acids, protein engineering of these homologous enzymes has enabled incorporation of over 100 different unnatural amino acids with diverse chemical and physical properties (Brown et al. 2018; Chin 2017; Nodling et al. 2019).

Despite these advances, the use of the amber codon to encode an unnatural amino acid is not without limitation. The amber codon can still be recognized by the release factor, causing translation termination and production of the truncated protein product. To circumvent this problem, different approaches have been explored. In one instance, an orthogonal ribosome has been engineered that has much lower affinity to the release factor (An and Chin 2009; Barrett and Chin 2010; Neumann et al. 2010). In fact, the orthogonal ribosome recognizes an alternative Kozak sequence, the amber codon on the reporter gene can be preferentially decoded as the unnatural amino acid, minimizing unwanted premature termination. In other approaches, the elongation factor can be engineered or the release factor can be removed to increase the amber suppression efficiency (Gan et al. 2017; Johnson et al. 2011; Schmied et al. 2014). Nevertheless, the general applicability of these approaches (An and Chin 2009; Barrett and Chin 2010; Gan et al. 2017; Johnson et al. 2011; Neumann et al. 2010; Schmied et al. 2014) is limited because the translational systems in prokaryotic cells and eukaryotic cells are vastly different and not readily interchangeable. Hence, an alternative approach that would function in both *E. coli* and mammalian cells is highly desirable, and this may be achieved by directly engineering the non-substrate binding part of the orthogonal synthetase to improve its catalytic activity (Owens et al. 2017; Sharma et al. 2018).

To improve unnatural amino acid incorporation, both MbPylRS and MmPylRS need to be thoroughly investigated. These homologs contain two domains, the C-terminal and N-terminal domains. The C-terminal domain possesses the catalytic site and binds to ATP and the unnatural amino acid. On the other hand, the N-terminal domain is important for the enzymatic activity in cells through interaction with the tRNA though is not directly involved in unnatural amino acid recognition (Suzuki et al. 2017). Often discovered through rational design, directed evolution or a combination of both, mutations in the C-terminal domain can expand the substrate scope, enabling incorporation of structurally diverse unnatural amino acids. On the other hand, though not directly involved in catalysis, mutations in the N-terminal domain have been demonstrated to affect the efficiency of unnatural amino acid incorporation in variants of MmPylRS, MbPylRS and their chimera (Bryson et al. 2017; Owens et al. 2017; Sharma et al. 2018). In a study to increase the incorporation of crotonyl lysine by a MbPylRS variant, six mutations (V8E, T13I, I36V, H45L, S121R, I355T) predominantly localized in the N-terminal domain were identified through random mutagenesis, and about threefold increase in crotonyl lysine incorporation was observed (Owens et al. 2017). When transferring these six mutations to another MbPylRS variant for incorporating *N*^*ε*^-[(2-propynyloxy)carbonyl]-L-lysine, a similar beneficial effect was observed. Another study found that R19H/H29R/T122S mutations in the N-terminal domain improved the capability of three MmPylRS variants for unnatural amino acid incorporation, and up to sixfold increase in the yields of recombinant proteins containing an unnatural amino acid was observed (Sharma et al. 2018). These studies indicate that mutations in the N-terminal domain can affect the incorporation efficiency. However, while C-terminal mutations are transferable between MbPylRS and MmPylRS to recognize specific unnatural amino acids (Brown et al. 2018; Chin 2017; Nodling et al. 2019), it remains elusive if the N-terminal mutations are also transferable between these two homologs.

**Fig. 1:**
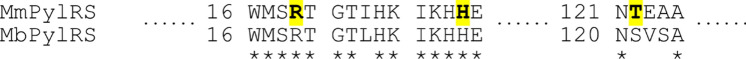
Partial sequence alignment of MmPylRS and MbPylRS. Amino acid sequence of MmPylRS (UniProt: Q8PWY1) and MbPylRS (UniProt: Q6WRH6) is aligned using ExPASy (https://web.expasy.org/sim/)

Provided that MbPylRS and MmPylRS are often used interchangeably for unnatural amino acid incorporation (Brown et al. 2018; Chin 2017; Nodling et al. 2019), beneficial mutations found in the N-terminal domain of one homolog are likely to be transferable to that of the other. As the MmPylRS variant R19H/H29R/T122S was reported to have improved activity, we propose that these mutations may have beneficial effect for the incorporation of unnatural amino acids by MbPylRS. As the corresponding position of MmPylRS T122 in MbPylRS is already a serine residue (Fig. 1), whereas R19 and H29 are conserved in the two homologs. Thus, we set out to probe the effect of R19H/H29R mutations in unnatural amino acid incorporation by MbPylRS variants.

**Fig. 2:**
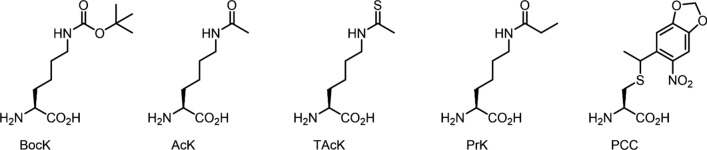
Structures of the five unnatural amino acids used in this study

Here, we examined four MbPylRS variants (i.e., wild-type, AcKRS, PrKRS, PCCRS) for incorporating unnatural amino acids (Fig. 2) under two different temperatures (37 °C or 25 °C). Five different unnatural amino acids *N*^*ε*^-Boc-lysine (BocK), acetyl lysine (AcK), thioacetyl lysine (TAcK), propionyl lysine (PrK) and photocaged cysteine (PCC) were used here for our examination. BocK can be incorporated by wild-type MbPylRS and is often used as a model unnatural amino acid in proof-of-principle studies. Both AcK and PrK are commonly used to investigate the effect of lysine post-translational modifications (i.e., lysine acetylation and propionylation, respectively) in bacteria and eukaryotic cells (Drazic et al. 2016; Ju and He 2017; Lin et al. 2012). TAcK is a stable analog mimicking lysine acetylation, but the carbonyl functionality is replaced with a thiocarbonyl group thereby preventing enzymatic hydrolysis (Venkat et al. 2017; Xiong et al. 2016). Due to the structural similarity, AcK and PrK can be incorporated by both AcKRS and PrKRS, and TAcK is likely to be accepted by these PylRS variants as well. On the other hand, PCC has a distinct structure when compared to the lysine analogs. PCC has a photolabile protecting group on the cysteine side chain, useful in light-controlled protein activation of active cysteines (Nguyen et al. 2014).

Overall, we found that R19H/H29R mutations have negligible benefits to MbPylRS variants. In two cases (i.e., PrKRS and PCCRS), the N-terminal mutations greatly decreased the unnatural amino acid incorporation. Our results highlight the difference between MbPylRS and MmPylRS, two closely related homologs, and raise concerns of using these homologs interchangeably in genetic code expansion.

## Methods

### Unnatural amino acids

BocK (Fluorochem, #078,520) and AcK (Fluorochem, #324,288) are commercially available. TAcK (Venkat et al. 2017), PrK (Gattner et al. 2013) and PCC (Nguyen et al. 2014) were synthesized following the literature procedures.

### Cloning

A MbPylRS variant and the Pyl tRNA are expressed from a pCDF vector, in which the synthetase and the tRNA are under the control of constitutively active GlnS promoter and Ipp promoter, respectively. Four variants of MbPylRS were tested here, including the wild-type enzyme, MbAcKRS (D76G/S123G/L266M/L270I/Y271F/L274A/C313F) (Neumann et al. 2009), MbPrKRS (Y271F/C313T) (Wilkins et al. 2015) and MbPCCRS (N311Q/C313A/V366M) (Nguyen et al. 2014). Apart from the wild-type enzyme, the other variants contain mutations indicated in the brackets for recognizing the designated unnatural amino acid (Table 1).

**Table 1:** Comparison of C-terminal mutations in the four MbPylRS variants used in this study

Residue no.	76	123	266	270	271	274	311	313	366
MbPylRS	D	S	L	L	Y	L	N	C	V
MbAcKRS	G	G	M	I	F	A	N	F	V
MbPrKRS	D	S	L	L	F	L	N	T	V
MbPCCRS	D	S	L	L	Y	L	Q	A	M

Plasmids PylST (Neumann et al. 2010), AcKST (Neumann et al. 2009) and PCCST (Nguyen et al. 2014) are kind gifts from Jason Chin. Plasmid PrKST was constructed by two rounds of PCR. Firstly, Y271F mutation was introduced using PylST as the template and the primers AACTATCTGCGTAAACTGGATCGTATTCTG and TCCAGTTTACGCAGATAGTTAAACAGGGTCGGGGCCAGCATC for PCR. The resulting PCR product (6 kb) was directly transformed into *E. coli* Stbl3 (ThermoFisher, #C737303), and plasmids were extracted from cells surviving in spectinomycin (50 µg/ml) containing media. The extracted plasmid served as the template for the second round of PCR with primers AATTCACCATGGTTAACTTTACCCAAATGGGCAGCGGCTGCAC and AAAGTTAACCATGGTGAATTCTTCCAGGTG to introduce mutation C313T, providing plasmid PrKST.

The N-terminal mutations (R19H/H29R) were introduced into each PylRS variant by PCR using primers TGGATGAGCCATACCGGCACCCTGCATAAAATCAAACATCGTGAAGTGAG and TGCCGGTATGGCTCATCCACAGGCCGGTCGCGCTAATCAGCACATC. All PCR reactions were performed using PrimeStar Max DNA polymerase (TaKaRa, #R045A).

Plasmid pET sfGFP(150TAG) has been deposited at the Addgene (Plasmid #133,455) and was constructed in two steps. Plasmid pBAD sfGFP (Reddington et al. 2012), a kind gift from Dafydd Jones, was digested with restriction enzyme NcoI and XhoI to excise the gene encoding sfGFP, which was cloned into a pET 28a vector using the same restriction sites to afford pET sfGFP. The mutation N150TAG was introduced by PCR using primers AATATAAATTCAACAGCCATTAGGTGTATATTACCGATAAACAG and TGGCTGTTGAAATTATATTCCAGTTTATGACC.

### Determining unnatural amino acid incorporation efficiency

**Fig. 3:**
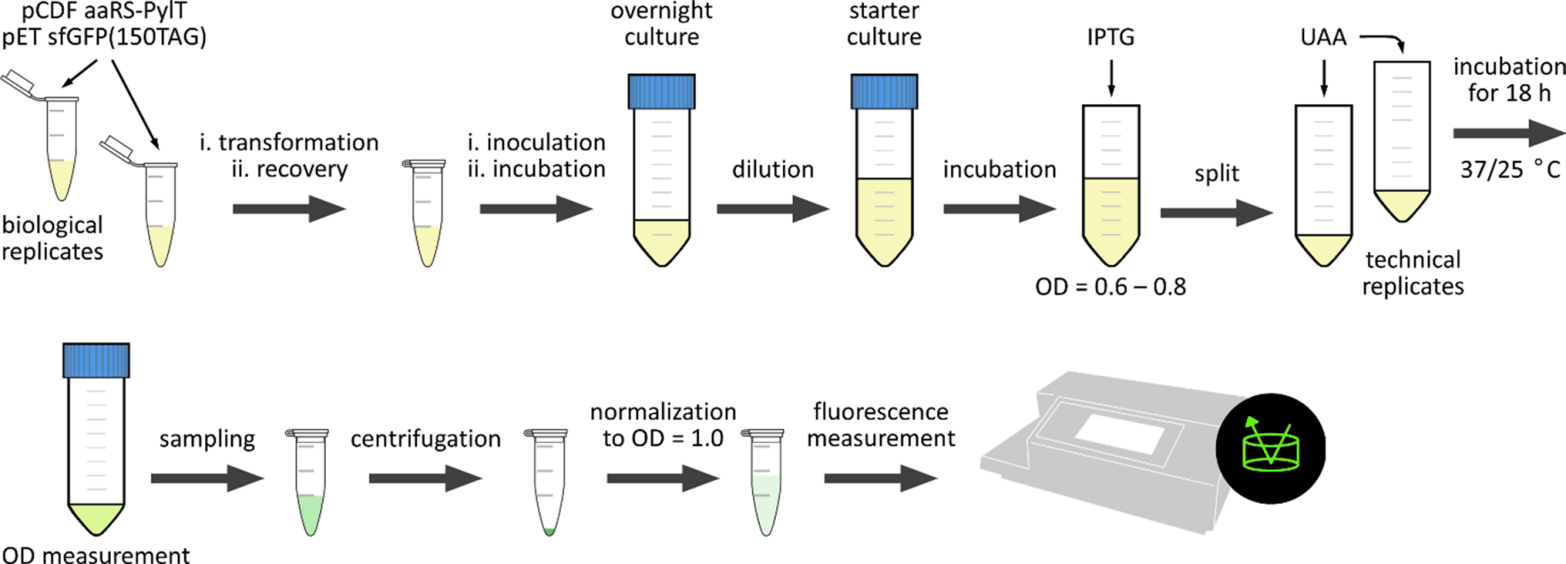
Experimental workflow. Cells were incubated at 37 °C unless otherwise stated. Biological replicates were prepared from independent transformations. Technical replicates were prepared from the IPTG-induced cultures. See text description for details

The workflow is illustrated in Fig. 3. Specifically, chemically competent *E. coli* BL21(DE3) cells were transformed with the reporter plasmid, pET sfGFP(150TAG), and the appropriate PylRS plasmid. The transformation was conducted by incubation with plasmids on ice for 10 min, heat shock at 42 °C for 45 s, incubation on ice for 2 min, and recovery in fresh LB media at 37 °C for 1 h with constant agitation. Cells were added into fresh LB media (10 ml) containing kanamycin (50 µg/ml, selection marker of the reporter plasmid) and spectinomycin (50 µg/ml, selection marker of the PylRS plasmid). Cells were cultured at 37 °C overnight with constant agitation.

The overnight culture was diluted into fresh LB media (25 ml) containing kanamycin (50 µg/ml) and spectinomycin (50 µg/ml) to reach OD_600_ = 0.1. This culture was incubated at 37 °C with constant agitation until OD_600_ = 0.6–0.8, and isopropyl β-D-1-thiogalactopyranoside (IPTG) was added to reach the final concentration of 1 mM. The culture was split into 50-ml falcon tubes (5-ml culture per tube), and unnatural amino acid was added, if required, to reach the final concentration of 5 mM (except for BocK, final concentration = 1 mM). Cultures were incubated at 25 °C or 37 °C for 18 h with constant agitation for protein production.

After incubation, OD_600_ of the cultures were recorded, and samples (1 ml) were taken from each culture. The samples were centrifuged at 20 °C and 21,130 × *g* for 5 min. Supernatants were removed, and the pellets were resuspended in PBS. The samples were centrifuged again at 20 °C and 21,130 × *g* for 5 min. Supernatants were removed, and the pellets were resuspended in PBS to OD_600_ = 1.0. The resuspension (200 µl) was placed into a 96-well plate, and the fluorescence was measured by a BMG Labtech FLUOstar OPTIMA microplate reader (excitation wavelength = 485 nm, emission wavelength = 520 nm, 20 flashes per well, 0.2 position delay, gain = 1000, shaking for 30 s before plate reading at 25 °C). Three biological repeats of each expression condition and two technical repeats of each fluorescent measurement were performed.

## Results

Our aim is to examine if the beneficial effect of R19H/H29R mutations found in the study of MmPylRS (Sharma et al. 2018) is transferable to MbPylRS. To investigate, we employed a fluorescence-based assay. Synthetase variants containing additional R19H/H29R mutations in the N-terminal domain of PylRS are denoted with an asterisk (*). For example, MbPylRS refers to the synthetase with the wild-type sequence, whereas MbPylRS* refers to the synthetase containing R19H/H29R mutations with respect to the wild-type sequence. In our assay, production of full-length and functional sfGFP only takes place when the amber codon is successfully suppressed (i.e., decoded by the Pyl tRNA for unnatural amino acid incorporation). As the fluorescence intensity is proportional to the quantity of full-length, functional sfGFP, it provides a quantitative measure with regards to the unnatural amino acid incorporation.

**Fig. 4:**
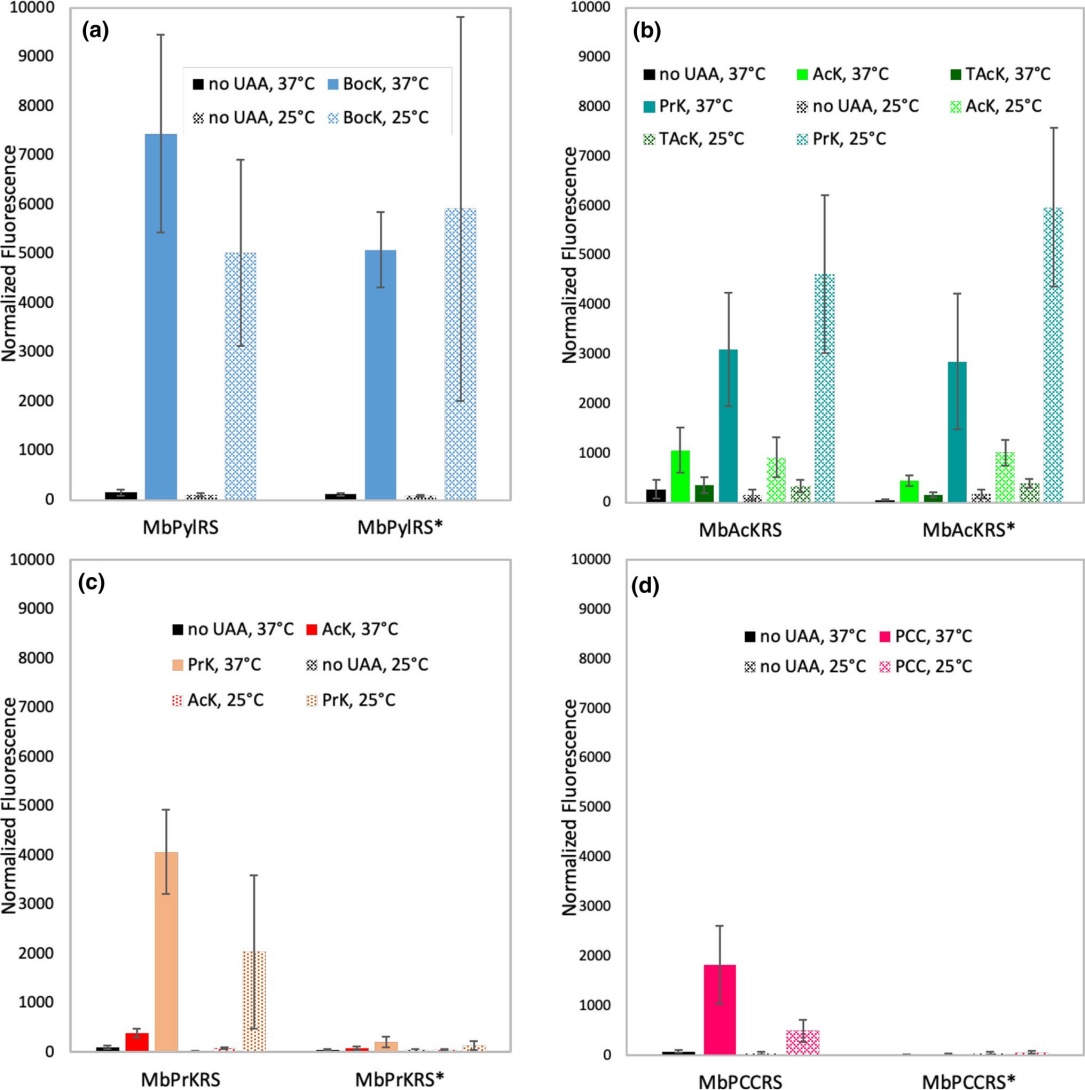
Unnatural amino acid incorporation efficiency by different MbPylRS variants. Transformed *E. coli* BL21(DE3) were normalized to OD_600_ = 1.0 before fluorescence measurement. Means and standard deviations are plotted, and their values are listed in Table 2. The asterisk (*) denotes synthetase variants contain additional R19H/H29R mutations in the N-terminal domain. UAA: unnatural amino acid. Fluorescence analysis of **a** wild-type MbPylRS, **b** AcKRS, **c** PrKRS and **d** PCCRS

In all MbPylRS synthetase variants tested, R19H/H29R mutations did not improve the incorporation efficiency over the wild-type sequence for any of the five amino acids (Fig. 4). At 25 °C, R19H/H29R mutations seemed to be beneficial to PylRS (Fig. 4a) and AcKRS (Fig. 4b) at the first glance. However, upon statistical analysis, the mean normalized fluorescence values for BocK incorporation by MbPylRS at 25 °C (Table 2, entry 2), as well as for AcK, TAcK or PrK incorporation by MbAcKRS at 25 °C (Table 2, entries 4, 6, 8) are not statistically significant different (*P* > 0.1) between the variants with the wild-type and the N-terminal mutations. Intriguingly, the N-terminal mutations greatly diminished the activity of PrKRS (Fig. 4c) and PCCRS (Fig. 4d). On the other hand, it is noteworthy that PrK is a better substrate than AcK in both PrKRS and AcKRS.

**Table 2:** *P* values of paired *t* tests comparing the effect of R19H/H29R in MbPylRS variants

Entry	Temperature	UAA	Normalized fluorescence (mean ± standard deviation)		*P* value
			Wild-type N-terminal	R19H/H29R	
1	37 °C	BocK	MbPylRS (7443 ± 2010)	MbPylRS* (5081 ± 764)	**1.0 × 10**^**–2**^
2	25 °C	BocK	MbPylRS (5023 ± 1895)	MbPylRS* (5919 ± 3900)	5.4 × 10^–1^
3	37 °C	AcK	MbAcKRS (1059 ± 459)	MbAcKRS* (443 ± 105)	**2.9 × 10**^**–3**^
4	25 °C	AcK	MbAcKRS (912 ± 403)	MbAcKRS* (1013 ± 255)	6.1 × 10^–1^
5	37 °C	TAcK	MbAcKRS (360 ± 165)	MbAcKRS* (157 ± 51)	**6.1 × 10**^**–3**^
6	25 °C	TAcK	MbAcKRS (339 ± 129)	MbAcKRS* (390 ± 83)	3.9 × 10^–1^
7	37 °C	PrK	MbAcKRS (3104 ± 1147)	MbAcKRS* (2851 ± 1375)	7.4 × 10^–1^
8	25 °C	PrK	MbAcKRS (4621 ± 1590)	MbAcKRS* (5972 ± 1610)	1.4 × 10^–1^
9	37 °C	AcK	MbPrKRS (384 ± 81)	MbPrKRS* (85 ± 38)	**8.3 × 10**^**–7**^
10	25 °C	AcK	MbPrKRS (77 ± 25)	MbPrKRS* (42 ± 13)	**1.3 × 10**^**–2**^
11	37 °C	PrK	MbPrKRS (4067 ± 853)	MbPrKRS* (203 ± 114)	**1.2 × 10**^**–7**^
12	25 °C	PrK	MbPrKRS (2039 ± 1556)	MbPrKRS* (135 ± 88)	**1.4 × 10**^**–2**^
13	37 °C	PCC	MbPCCRS (1828 ± 795)	MbPCCRS* (21 ± 8)	**1.2 × 10**^**–6**^
14	25 °C	PCC	MbPCCRS (494 ± 229)	MbPCCRS* (60 ± 26)	**1.0 × 10**^**–5**^

*P* values < 0.05 are shown in bold. Original fluorescence data are in S2 data

## Discussion

Here, we investigated the effect of R19H/H29R mutations in MbPylRS variants for unnatural amino acid incorporation. We tested four MbPylRS variants (i.e., wild-type, AcKRS, PrKRS, PCCRS), five unnatural amino acids (BocK, AcK, TAcK, PrK, PCC), and two culture temperatures (37 °C or 25 °C). While for three MmPylRS variants it was found that R19H/H29R mutations together with T122S mutation (corresponding residue of MmPyl Thr122 is already Ser, Fig. 1) improved unnatural amino acid incorporation (12), MbPylRS variants containing R19H/H29R mutations do not show any beneficial effect.

There is no correlation between point mutations in the C-terminus of the PylRS variants and the effect of N-terminal mutations on incorporation efficiency. This is evident by the lack of activity observed with MbPrKRS* and MbPCCRS*, negligible difference was observed between MbPylRS and MbPylRS*, as well as MbAcKRS and MbAcKRS*. It is likely that the two closely related homologs, while often used interchangeably in genetic code expansion (Brown et al. 2018; Chin 2017; Nodling et al. 2019), are different in their underlying molecular mechanisms for functional regulation and catalytic activity. Indeed, there are 35 extra amino acid residues in the N-terminal domain of MmPylRS, and this difference accounts for the difference in the length of the two homologs (MmPylRS: 454 amino acids; MbPylRS: 419 amino acids). On the other hand, wild-type MmPylRS and MbPylRS have been reported with different substrate scope. For example, while *N*^*ε*^-L-thiaprolyl-L-lysine and *N*^*ε*^-L-cysteinyl-L-lysine are barely recognized by wild-type MmPylRS as a substrate, they can be readily incorporated into a protein using wild-type MbPylRS (Nguyen et al. 2011). To improve unnatural amino acid incorporation efficiency, it would be necessary to perform detailed investigation of each homolog independently and caution should be taken when transferring mutations between MmPylRS and MbPylRS.

## Limitations

We have only tested the transferability of one set of N-terminal mutations between MmPylRS and MbPylRS.

## Supplementary Information

Below is the link to the electronic supplementary material.

Nucleotide and amino acid sequences of MbPylRS variants used in this study (DOCX 17 KB)

Original fluorescence data for unnatural amino acid incorporation efficiency by different MbPylRS variants. Methodological details are given in under Material and Methods section (XLSX 85 KB)

## Data Availability

Data are available in the two supplementary files.

## Abbreviations

AcK: Acetyl lysine
BocK: N^ε^-Boc-lysine
IPTG: Isopropyl β-d-1-thiogalactopyranoside
MbPylRS: *Methanosarcina barkeri* Pyrrolysyl-tRNA synthetase
MmPylRS: *Methanosarcina mazei* Pyrrolysyl-tRNA synthetase
PCC: Photocaged cysteine
PylRS: Pyrrolysyl-tRNA synthetase
PrK: Propionyl lysine
TAcK: Thioacetyl lysine
