# Supplementary material for: Transferability of N-terminal mutations of pyrrolysyl-tRNA synthetase in one species to that in another species on unnatural amino acid incorporation efficiency
Source: Amino Acids. 2020 Dec 17;53(1):89–96. doi: 10.1007/s00726-020-02927-z (PMC7822784; doi:10.1007/s00726-020-02927-z)
Supplement: Supplementary file 1 — Nucleotide and amino acid sequences of MbPylRS variants used in this study (DOCX 17 KB) [file 726_2020_2927_MOESM1_ESM.docx]

**Nucleotide and Amino Acid Sequences**

PylRS

atggataaaaaaccgctggatgtgctgattagcgcgaccggcctgtggatgagcCGTaccggcaccctgcataaaatcaaacatCATgaagtgagccgcagcaaaatctatattgaaatggcgtgcggcgatcatctggtggtgaacaacagccgtagctgccgtaccgcgcgtgcgtttcgtcatcataaataccgcaaaacctgcaaacgttgccgtgtgagcgatgaagatatcaacaactttctgacccgtagcaccgaaagcaaaaacagcgtgaaagtgcgtgtggtgagcgcgccgaaagtgaaaaaagcgatgccgaaaagcgtgagccgtgcgccgaaaccgctggaaaatagcgtgagcgcgaaagcgagcaccaacaccagccgtagcgttccgagcccggcgaaaagcaccccgaacagcagcgttccggcgtctgcgccggcaccgagcctgacccgcagccagctggatcgtgtggaagcgctgctgtctccggaagataaaattagcctgaacatggcgaaaccgtttcgtgaactggaaccggaactggtgacccgtcgtaaaaacgattttcagcgcctgtataccaacgatcgtgaagattatctgggcaaactggaacgtgatatcaccaaattttttgtggatcgcggctttctggaaattaaaagcccgattctgattccggcggaatatgtggaacgtatgggcattaacaacgacaccgaactgagcaaacaaattttccgcgtggataaaaacctgtgcctgcgtccgatgctggccccgaccctgtataactatctgcgtaaactggatcgtattctgccgggtccgatcaaaatttttgaagtgggcccgtgctatcgcaaagaaagcgatggcaaagaacacctggaagaattcaccatggttaacttttgccaaatgggcagcggctgcacccgtgaaaacctggaagcgctgatcaaagaattcctggattatctggaaatcgacttcgaaattgtgggcgatagctgcatggtgtatggcgataccctggatattatgcatggcgatctggaactgagcagcgcggtggtgggtccggttagcctggatcgtgaatggggcattgataaaccgtggattggcgcgggttttggcctggaacgtctgctgaaagtgatgcatggcttcaaaaacattaaacgtgcgagccgtagcgaaagctactataacggcattagcacgaacctgtaa

MDKKPLDVLISATGLWMSRTGTLHKIKHHEVSRSKIYIEMACGDHLVVNNSRSCRTARAFRHHKYRKTCKRCRVSDEDINNFLTRSTESKNSVKVRVVSAPKVKKAMPKSVSRAPKPLENSVSAKASTNTSRSVPSPAKSTPNSSVPASAPAPSLTRSQLDRVEALLSPEDKISLNMAKPFRELEPELVTRRKNDFQRLYTNDREDYLGKLERDITKFFVDRGFLEIKSPILIPAEYVERMGINNDTELSKQIFRVDKNLCLRPMLAPTLYNYLRKLDRILPGPIKIFEVGPCYRKESDGKEHLEEFTMVNFCQMGSGCTRENLEALIKEFLDYLEIDFEIVGDSCMVYGDTLDIMHGDLELSSAVVGPVSLDREWGIDKPWIGAGFGLERLLKVMHGFKNIKRASRSESYYNGISTNL*

PylRS*

atggataaaaaaccgctggatgtgctgattagcgcgaccggcctgtggatgagcCATaccggcaccctgcataaaatcaaacatCGTgaagtgagccgcagcaaaatctatattgaaatggcgtgcggcgatcatctggtggtgaacaacagccgtagctgccgtaccgcgcgtgcgtttcgtcatcataaataccgcaaaacctgcaaacgttgccgtgtgagcgatgaagatatcaacaactttctgacccgtagcaccgaaagcaaaaacagcgtgaaagtgcgtgtggtgagcgcgccgaaagtgaaaaaagcgatgccgaaaagcgtgagccgtgcgccgaaaccgctggaaaatagcgtgagcgcgaaagcgagcaccaacaccagccgtagcgttccgagcccggcgaaaagcaccccgaacagcagcgttccggcgtctgcgccggcaccgagcctgacccgcagccagctggatcgtgtggaagcgctgctgtctccggaagataaaattagcctgaacatggcgaaaccgtttcgtgaactggaaccggaactggtgacccgtcgtaaaaacgattttcagcgcctgtataccaacgatcgtgaagattatctgggcaaactggaacgtgatatcaccaaattttttgtggatcgcggctttctggaaattaaaagcccgattctgattccggcggaatatgtggaacgtatgggcattaacaacgacaccgaactgagcaaacaaattttccgcgtggataaaaacctgtgcctgcgtccgatgctggccccgaccctgtataactatctgcgtaaactggatcgtattctgccgggtccgatcaaaatttttgaagtgggcccgtgctatcgcaaagaaagcgatggcaaagaacacctggaagaattcaccatggttaacttttgccaaatgggcagcggctgcacccgtgaaaacctggaagcgctgatcaaagaattcctggattatctggaaatcgacttcgaaattgtgggcgatagctgcatggtgtatggcgataccctggatattatgcatggcgatctggaactgagcagcgcggtggtgggtccggttagcctggatcgtgaatggggcattgataaaccgtggattggcgcgggttttggcctggaacgtctgctgaaagtgatgcatggcttcaaaaacattaaacgtgcgagccgtagcgaaagctactataacggcattagcacgaacctgtaa

MDKKPLDVLISATGLWMSHTGTLHKIKHREVSRSKIYIEMACGDHLVVNNSRSCRTARAFRHHKYRKTCKRCRVSDEDINNFLTRSTESKNSVKVRVVSAPKVKKAMPKSVSRAPKPLENSVSAKASTNTSRSVPSPAKSTPNSSVPASAPAPSLTRSQLDRVEALLSPEDKISLNMAKPFRELEPELVTRRKNDFQRLYTNDREDYLGKLERDITKFFVDRGFLEIKSPILIPAEYVERMGINNDTELSKQIFRVDKNLCLRPMLAPTLYNYLRKLDRILPGPIKIFEVGPCYRKESDGKEHLEEFTMVNFCQMGSGCTRENLEALIKEFLDYLEIDFEIVGDSCMVYGDTLDIMHGDLELSSAVVGPVSLDREWGIDKPWIGAGFGLERLLKVMHGFKNIKRASRSESYYNGISTNL*

AcKRS

ATGGATAAAAAACCGCTGGATGTGCTGATTAGCGCGACCGGCCTGTGGATGAGCCGTACCGGCACCCTGCATAAAATCAAACATCATGAAGTGAGCCGCAGCAAAATCTATATTGAAATGGCGTGCGGCGATCATCTGGTGGTGAACAACAGCCGTAGCTGCCGTACCGCGCGTGCGTTTCGTCATCATAAATACCGCAAAACCTGCAAACGTTGCCGTGTGAGCGATGAAGATATCAACAACTTTCTGACCCGTAGCACCGAAAGCAAAAACAGCGTGAAAGTGCGTGTGGTGAGCGCGCCGAAAGTGAAAAAAGCGATGCCGAAAAGCGTGAGCCGTGCGCCGAAACCGCTGGAAAATAGCGTGAGCGCGAAAGCGAGCACCAACACCAGCCGTAGCGTTCCGAGCCCGGCGAAAAGCACCCCGAACAGCAGCGTTCCGGCGTCTGCGCCGGCACCGAGCCTGACCCGCAGCCAGCTGGATCGTGTGGAAGCGCTGCTGTCTCCGGAAGATAAAATTAGCCTGAACATGGCGAAACCGTTTCGTGAACTGGAACCGGAACTGGTGACCCGTCGTAAAAACGATTTTCAGCGCCTGTATACCAACGATCGTGAAGATTATCTGGGCAAACTGGAACGTGATATCACCAAATTTTTTGTGGATCGCGGCTTTCTGGAAATTAAAAGCCCGATTCTGATTCCGGCGGAATATGTGGAACGTATGGGCATTAACAACGACACCGAACTGAGCAAACAAATTTTCCGCGTGGATAAAAACCTGTGCCTGCGTCCGATGaTGGCtCCGACCaTtTtTAACTATgctCGTAAACTGGATCGTATTCTGCCGGGTCCGATCAAAATTTTTGAAGTGGGCCCGTGCTATCGCAAAGAAAGCGATGGCAAAGAACACCTGGAAGAATTCACCATGGTTaacTTTtttCAAATGGGCAGCGGCTGCACCCGTGAAAACCTGGAAGCGCTGATCAAAGAATTCCTGGATTATCTGGAAATCGACTTCGAAATTGTGGGCGATAGCTGCATGGTGTATGGCGATACCCTGGATATTATGCATGGCGATCTGGAACTGAGCAGCGCGgtgGTGGGTCCGGTTAGCCTGGATCGTGAATGGGGCATTGATAAACCGtggATTGGCGCGggtTTTGGCCTGGAACGTCTGCTGAAAGTGATGCATGGCTTCAAAAACATTAAACGTGCGAGCCGTAGCGAAAGCTACTATAACGGCATTAGCACGAACCTGTAA

MMDKKPLDVLISATGLWMSRTGTLHKIKHHEVSRSKIYIEMACGDHLVVNNSRSCRTARAFRHHKYRKTCKRCRVSGEDINNFLTRSTESKNSVKVRVVSAPKVKKAMPKSVSRAPKPLENSVGAKASTNTSRSVPSPAKSTPNSSVPASAPAPSLTRSQLDRVEALLSPEDKISLNMAKPFRELEPELVTRRKNDFQRLYTNDREDYLGKLERDITKFFVDRGFLEIKSPILIPAEYVERMGINNDTELSKQIFRVDKNLCLRPMMAPTIFNYARKLDRILPGPIKIFEVGPCYRKESDGKEHLEEFTMVNFFQMGSGCTRENLEALIKEFLDYLEIDFEIVGDSCMVYGDTLDIMHGDLELSSAVVGPVSLDREWGIDKPWIGAGFGLERLLKVMHGFKNIKRASRSESYYNGISTNL*

AcKRS*

ATGATGGATAAAAAACCGCTGGATGTGCTGATTAGCGCGACCGGCCTGTGGATGAGCCATACCGGCACCCTGCATAAAATCAAACATCGTGAAGTGAGCCGCAGCAAAATCTATATTGAAATGGCGTGCGGCGATCATCTGGTGGTGAACAACAGCCGTAGCTGCCGTACCGCGCGTGCGTTTCGTCATCATAAATACCGCAAAACCTGCAAACGTTGCCGTGTGAGCGATGAAGATATCAACAACTTTCTGACCCGTAGCACCGAAAGCAAAAACAGCGTGAAAGTGCGTGTGGTGAGCGCGCCGAAAGTGAAAAAAGCGATGCCGAAAAGCGTGAGCCGTGCGCCGAAACCGCTGGAAAATAGCGTGAGCGCGAAAGCGAGCACCAACACCAGCCGTAGCGTTCCGAGCCCGGCGAAAAGCACCCCGAACAGCAGCGTTCCGGCGTCTGCGCCGGCACCGAGCCTGACCCGCAGCCAGCTGGATCGTGTGGAAGCGCTGCTGTCTCCGGAAGATAAAATTAGCCTGAACATGGCGAAACCGTTTCGTGAACTGGAACCGGAACTGGTGACCCGTCGTAAAAACGATTTTCAGCGCCTGTATACCAACGATCGTGAAGATTATCTGGGCAAACTGGAACGTGATATCACCAAATTTTTTGTGGATCGCGGCTTTCTGGAAATTAAAAGCCCGATTCTGATTCCGGCGGAATATGTGGAACGTATGGGCATTAACAACGACACCGAACTGAGCAAACAAATTTTCCGCGTGGATAAAAACCTGTGCCTGCGTCCGATGaTGGCtCCGACCaTtTtTAACTATgctCGTAAACTGGATCGTATTCTGCCGGGTCCGATCAAAATTTTTGAAGTGGGCCCGTGCTATCGCAAAGAAAGCGATGGCAAAGAACACCTGGAAGAATTCACCATGGTTaacTTTtttCAAATGGGCAGCGGCTGCACCCGTGAAAACCTGGAAGCGCTGATCAAAGAATTCCTGGATTATCTGGAAATCGACTTCGAAATTGTGGGCGATAGCTGCATGGTGTATGGCGATACCCTGGATATTATGCATGGCGATCTGGAACTGAGCAGCGCGgtgGTGGGTCCGGTTAGCCTGGATCGTGAATGGGGCATTGATAAACCGtggATTGGCGCGggtTTTGGCCTGGAACGTCTGCTGAAAGTGATGCATGGCTTCAAAAACATTAAACGTGCGAGCCGTAGCGAAAGCTACTATAACGGCATTAGCACGAACCTGTAA

MMDKKPLDVLISATGLWMSHTGTLHKIKHREVSRSKIYIEMACGDHLVVNNSRSCRTARAFRHHKYRKTCKRCRVSDEDINNFLTRSTESKNSVKVRVVSAPKVKKAMPKSVSRAPKPLENSVSAKASTNTSRSVPSPAKSTPNSSVPASAPAPSLTRSQLDRVEALLSPEDKISLNMAKPFRELEPELVTRRKNDFQRLYTNDREDYLGKLERDITKFFVDRGFLEIKSPILIPAEYVERMGINNDTELSKQIFRVDKNLCLRPMMAPTIFNYARKLDRILPGPIKIFEVGPCYRKESDGKEHLEEFTMVNFFQMGSGCTRENLEALIKEFLDYLEIDFEIVGDSCMVYGDTLDIMHGDLELSSAVVGPVSLDREWGIDKPWIGAGFGLERLLKVMHGFKNIKRASRSESYYNGISTNL*

PrKRS

atggataaaaaaccgctggatgtgctgattagcgcgaccggcctgtggatgagccgtaccggcaccctgcataaaatcaaacatcatgaagtgagccgcagcaaaatctatattgaaatggcgtgcggcgatcatctggtggtgaacaacagccgtagctgccgtaccgcgcgtgcgtttcgtcatcataaataccgcaaaacctgcaaacgttgccgtgtgagcgatgaagatatcaacaactttctgacccgtagcaccgaaagcaaaaacagcgtgaaagtgcgtgtggtgagcgcgccgaaagtgaaaaaagcgatgccgaaaagcgtgagccgtgcgccgaaaccgctggaaaatagcgtgagcgcgaaagcgagcaccaacaccagccgtagcgttccgagcccggcgaaaagcaccccgaacagcagcgttccggcgtctgcgccggcaccgagcctgacccgcagccagctggatcgtgtggaagcgctgctgtctccggaagataaaattagcctgaacatggcgaaaccgtttcgtgaactggaaccggaactggtgacccgtcgtaaaaacgattttcagcgcctgtataccaacgatcgtgaagattatctgggcaaactggaacgtgatatcaccaaattttttgtggatcgcggctttctggaaattaaaagcccgattctgattccggcggaatatgtggaacgtatgggcattaacaacgacaccgaactgagcaaacaaattttccgcgtggataaaaacctgtgcctgcgtccgatgctggccccgaccctgTTTaactatctgcgtaaactggatcgtattctgccgggtccgatcaaaatttttgaagtgggcccgtgctatcgcaaagaaagcgatggcaaagaacacctggaagaattcaccatggttaactttACCcaaatgggcagcggctgcacccgtgaaaacctggaagcgctgatcaaagaattcctggattatctggaaatcgacttcgaaattgtgggcgatagctgcatggtgtatggcgataccctggatattatgcatggcgatctggaactgagcagcgcggtggtgggtccggttagcctggatcgtgaatggggcattgataaaccgtggattggcgcgggttttggcctggaacgtctgctgaaagtgatgcatggcttcaaaaacattaaacgtgcgagccgtagcgaaagctactataacggcattagcacgaacctgtaa

MDKKPLDVLISATGLWMSRTGTLHKIKHHEVSRSKIYIEMACGDHLVVNNSRSCRTARAFRHHKYRKTCKRCRVSDEDINNFLTRSTESKNSVKVRVVSAPKVKKAMPKSVSRAPKPLENSVSAKASTNTSRSVPSPAKSTPNSSVPASAPAPSLTRSQLDRVEALLSPEDKISLNMAKPFRELEPELVTRRKNDFQRLYTNDREDYLGKLERDITKFFVDRGFLEIKSPILIPAEYVERMGINNDTELSKQIFRVDKNLCLRPMLAPTLFNYLRKLDRILPGPIKIFEVGPCYRKESDGKEHLEEFTMVNFTQMGSGCTRENLEALIKEFLDYLEIDFEIVGDSCMVYGDTLDIMHGDLELSSAVVGPVSLDREWGIDKPWIGAGFGLERLLKVMHGFKNIKRASRSESYYNGISTNL*

PrKRS*

atggataaaaaaccgctggatgtgctgattagcgcgaccggcctgtggatgagccAtaccggcaccctgcataaaatcaaacatCGTgaagtgagccgcagcaaaatctatattgaaatggcgtgcggcgatcatctggtggtgaacaacagccgtagctgccgtaccgcgcgtgcgtttcgtcatcataaataccgcaaaacctgcaaacgttgccgtgtgagcgatgaagatatcaacaactttctgacccgtagcaccgaaagcaaaaacagcgtgaaagtgcgtgtggtgagcgcgccgaaagtgaaaaaagcgatgccgaaaagcgtgagccgtgcgccgaaaccgctggaaaatagcgtgagcgcgaaagcgagcaccaacaccagccgtagcgttccgagcccggcgaaaagcaccccgaacagcagcgttccggcgtctgcgccggcaccgagcctgacccgcagccagctggatcgtgtggaagcgctgctgtctccggaagataaaattagcctgaacatggcgaaaccgtttcgtgaactggaaccggaactggtgacccgtcgtaaaaacgattttcagcgcctgtataccaacgatcgtgaagattatctgggcaaactggaacgtgatatcaccaaattttttgtggatcgcggctttctggaaattaaaagcccgattctgattccggcggaatatgtggaacgtatgggcattaacaacgacaccgaactgagcaaacaaattttccgcgtggataaaaacctgtgcctgcgtccgatgctggccccgaccctgTTTaactatctgcgtaaactggatcgtattctgccgggtccgatcaaaatttttgaagtgggcccgtgctatcgcaaagaaagcgatggcaaagaacacctggaagaattcaccatggttaactttACCcaaatgggcagcggctgcacccgtgaaaacctggaagcgctgatcaaagaattcctggattatctggaaatcgacttcgaaattgtgggcgatagctgcatggtgtatggcgataccctggatattatgcatggcgatctggaactgagcagcgcggtggtgggtccggttagcctggatcgtgaatggggcattgataaaccgtggattggcgcgggttttggcctggaacgtctgctgaaagtgatgcatggcttcaaaaacattaaacgtgcgagccgtagcgaaagctactataacggcattagcacgaacctgtaa

MDKKPLDVLISATGLWMSHTGTLHKIKHREVSRSKIYIEMACGDHLVVNNSRSCRTARAFRHHKYRKTCKRCRVSDEDINNFLTRSTESKNSVKVRVVSAPKVKKAMPKSVSRAPKPLENSVSAKASTNTSRSVPSPAKSTPNSSVPASAPAPSLTRSQLDRVEALLSPEDKISLNMAKPFRELEPELVTRRKNDFQRLYTNDREDYLGKLERDITKFFVDRGFLEIKSPILIPAEYVERMGINNDTELSKQIFRVDKNLCLRPMLAPTLFNYLRKLDRILPGPIKIFEVGPCYRKESDGKEHLEEFTMVNFTQMGSGCTRENLEALIKEFLDYLEIDFEIVGDSCMVYGDTLDIMHGDLELSSAVVGPVSLDREWGIDKPWIGAGFGLERLLKVMHGFKNIKRASRSESYYNGISTNL*

PCCRS

ATGGATAAAAAACCGCTGGATGTGCTGATTAGCGCGACCGGCCTGTGGATGAGCCGTACCGGCACCCTGCATAAAATCAAACATCATGAAGTGAGCCGCAGCAAAATCTATATTGAAATGGCGTGCGGCGATCATCTGGTGGTGAACAACAGCCGTAGCTGCCGTACCGCGCGTGCGTTTCGTCATCATAAATACCGCAAAACCTGCAAACGTTGCCGTGTGAGCGATGAAGATATCAACAACTTTCTGACCCGTAGCACCGAAAGCAAAAACAGCGTGAAAGTGCGTGTGGTGAGCGCGCCGAAAGTGAAAAAAGCGATGCCGAAAAGCGTGAGCCGTGCGCCGAAACCGCTGGAAAATAGCGTGAGCGCGAAAGCGAGCACCAACACCAGCCGTAGCGTTCCGAGCCCGGCGAAAAGCACCCCGAACAGCAGCGTTCCGGCGTCTGCGCCGGCACCGAGCCTGACCCGCAGCCAGCTGGATCGTGTGGAAGCGCTGCTGTCTCCGGAAGATAAAATTAGCCTGAACATGGCGAAACCGTTTCGTGAACTGGAACCGGAACTGGTGACCCGTCGTAAAAACGATTTTCAGCGCCTGTATACCAACGATCGTGAAGATTATCTGGGCAAACTGGAACGTGATATCACCAAATTTTTTGTGGATCGCGGCTTTCTGGAAATTAAAAGCCCGATTCTGATTCCGGCGGAATATGTGGAACGTATGGGCATTAACAACGACACCGAACTGAGCAAACAAATTTTCCGCGTGGATAAAAACCTGTGCCTGCGTCCGATGCTGGCCCCGACCCTGTATAACTATCTGCGTAAACTGGATCGTATTCTGCCGGGTCCGATCAAAATTTTTGAAGTGGGCCCGTGCTATCGCAAAGAAAGCGATGGCAAAGAACACCTGGAAGAATTCACCATGGTTcagTTTgcgCAAATGGGCAGCGGCTGCACCCGTGAAAACCTGGAAGCGCTGATCAAgGAATTCCTGGATTATCTGGAAATCGACTTCGAAATTGTGGGCGATAGCTGCATGGTGTATGGCGATACCCTGGATATTATGCATGGCGATCTGGAACTGAGCAGCGCGatgGTGGGTCCGGTTAGCCTGGATCGTGAATGGGGCATTGATAAACCGtggATTGGCGCGgggTTTGGCCTGGAACGTCTGCTGAAAGTGATGCATGGCTTCAAAAACATTAAACGTGCGAGCCGTAGCGAAAGCTACTATAACGGCATTAGCACGAACCTGtaa

MDKKPLDVLISATGLWMSRTGTLHKIKHHEVSRSKIYIEMACGDHLVVNNSRSCRTARAFRHHKYRKTCKRCRVSDEDINNFLTRSTESKNSVKVRVVSAPKVKKAMPKSVSRAPKPLENSVSAKASTNTSRSVPSPAKSTPNSSVPASAPAPSLTRSQLDRVEALLSPEDKISLNMAKPFRELEPELVTRRKNDFQRLYTNDREDYLGKLERDITKFFVDRGFLEIKSPILIPAEYVERMGINNDTELSKQIFRVDKNLCLRPMLAPTLYNYLRKLDRILPGPIKIFEVGPCYRKESDGKEHLEEFTMVQFAQMGSGCTRENLEALIKEFLDYLEIDFEIVGDSCMVYGDTLDIMHGDLELSSAMVGPVSLDREWGIDKPWIGAGFGLERLLKVMHGFKNIKRASRSESYYNGISTNL*

PCCRS*

ATGGATAAAAAACCGCTGGATGTGCTGATTAGCGCGACCGGCCTGTGGATGAGCCATACCGGCACCCTGCATAAAATCAAACATCGTGAAGTGAGCCGCAGCAAAATCTATATTGAAATGGCGTGCGGCGATCATCTGGTGGTGAACAACAGCCGTAGCTGCCGTACCGCGCGTGCGTTTCGTCATCATAAATACCGCAAAACCTGCAAACGTTGCCGTGTGAGCGATGAAGATATCAACAACTTTCTGACCCGTAGCACCGAAAGCAAAAACAGCGTGAAAGTGCGTGTGGTGAGCGCGCCGAAAGTGAAAAAAGCGATGCCGAAAAGCGTGAGCCGTGCGCCGAAACCGCTGGAAAATAGCGTGAGCGCGAAAGCGAGCACCAACACCAGCCGTAGCGTTCCGAGCCCGGCGAAAAGCACCCCGAACAGCAGCGTTCCGGCGTCTGCGCCGGCACCGAGCCTGACCCGCAGCCAGCTGGATCGTGTGGAAGCGCTGCTGTCTCCGGAAGATAAAATTAGCCTGAACATGGCGAAACCGTTTCGTGAACTGGAACCGGAACTGGTGACCCGTCGTAAAAACGATTTTCAGCGCCTGTATACCAACGATCGTGAAGATTATCTGGGCAAACTGGAACGTGATATCACCAAATTTTTTGTGGATCGCGGCTTTCTGGAAATTAAAAGCCCGATTCTGATTCCGGCGGAATATGTGGAACGTATGGGCATTAACAACGACACCGAACTGAGCAAACAAATTTTCCGCGTGGATAAAAACCTGTGCCTGCGTCCGATGCTGGCCCCGACCCTGTATAACTATCTGCGTAAACTGGATCGTATTCTGCCGGGTCCGATCAAAATTTTTGAAGTGGGCCCGTGCTATCGCAAAGAAAGCGATGGCAAAGAACACCTGGAAGAATTCACCATGGTTcagTTTgcgCAAATGGGCAGCGGCTGCACCCGTGAAAACCTGGAAGCGCTGATCAAgGAATTCCTGGATTATCTGGAAATCGACTTCGAAATTGTGGGCGATAGCTGCATGGTGTATGGCGATACCCTGGATATTATGCATGGCGATCTGGAACTGAGCAGCGCGatgGTGGGTCCGGTTAGCCTGGATCGTGAATGGGGCATTGATAAACCGtggATTGGCGCGgggTTTGGCCTGGAACGTCTGCTGAAAGTGATGCATGGCTTCAAAAACATTAAACGTGCGAGCCGTAGCGAAAGCTACTATAACGGCATTAGCACGAACCTGTAA

MDKKPLDVLISATGLWMSHTGTLHKIKHREVSRSKIYIEMACGDHLVVNNSRSCRTARAFRHHKYRKTCKRCRVSDEDINNFLTRSTESKNSVKVRVVSAPKVKKAMPKSVSRAPKPLENSVSAKASTNTSRSVPSPAKSTPNSSVPASAPAPSLTRSQLDRVEALLSPEDKISLNMAKPFRELEPELVTRRKNDFQRLYTNDREDYLGKLERDITKFFVDRGFLEIKSPILIPAEYVERMGINNDTELSKQIFRVDKNLCLRPMLAPTLYNYLRKLDRILPGPIKIFEVGPCYRKESDGKEHLEEFTMVQFAQMGSGCTRENLEALIKEFLDYLEIDFEIVGDSCMVYGDTLDIMHGDLELSSAMVGPVSLDREWGIDKPWIGAGFGLERLLKVMHGFKNIKRASRSESYYNGISTNL*
